# Long-term deep-TMS does not negatively affect cognitive functions in stroke and spinal cord injury patients with central neuropathic pain

**DOI:** 10.1186/s12883-019-1531-z

**Published:** 2019-12-10

**Authors:** Priscila Mara Lorencini Selingardi, Antonia Lilian de Lima Rodrigues, Valquíria Aparecida da Silva, Diego Toledo Reis Mendes Fernandes, Jefferson Rosí, Marco Antônio Marcolin, Lin T. Yeng, André R. Brunoni, Manoel J. Teixeira, Ricardo Galhardoni, Daniel Ciampi de Andrade

**Affiliations:** 1 Pain Center, LIM 62 Neurosurgery, Department of Neurology, University of São Paulo, São Paulo, Brazil; 2 Pain Center, Instituto do Câncer do Estado de São Paulo, São Paulo, Brazil; 3 Service of Interdisciplinary Neuromodulation (SIN), Laboratory of Neuroscience (LIM27) and National Institute of Biomarkers in Neuropsychiatry (INBioN), Department and Institute of Psychiatry, University of São Paulo School of Medicine, São Paulo, Brazil; 4 School of Medicine, University of City of São Paulo (UNICID), São Paulo, Brazil; 5 Department of Internal Medicine, University Hospital, University of São Paulo, São Paulo, Brazil; 6 Divisão de Clínica Neurocirúrgica do Hospital das Clínicas da FMUSP, Instituto Central, Av. Dr. Enéas de Carvalho Aguiar, 255, 5˚ andar, sala 5084, Cerqueira César, São Paulo, 05403-900 Brazil

## Background

Conventional superficial transcranial magnetic stimulation (s-TMS) has been studied for the treatment of several neuropsychiatric disorders for the last two decades [1]. It entered the armamentarium against major depression in the US in 2008 [2] and is currently clinically used for the relief of the non-motor symptoms of Parkinson’s disease and of chronic pain [3], as well as for the pre-operative identification of responders before implantable epidural cortical stimulation for refractory neuropathic pain [4]. During the first years of sTMS use in clinical practice, the risk of seizures was the main adverse events-related concern, and several safety guidelines were published to screen for increased risk of seizures and to mitigate its occurrence [5]. With the accumulation of studies attesting to the low risk of seizures after repetitive transcranial magnetic stimulation (rTMS) when recommended safety criteria are followed, the focus of safety-related preoccupations has shifted towards the potential long-term cognitive and behavioral effects of sTMS protocols. Because several targets of s-TMS studies play an important whole in cognitive performance [6], the excitation or inhibition of these cortical areas could impair the patient’s performance in certain cognitive domains. This is potentially important if one acknowledges that several neuropsychiatric disorders treated by transcranial magnetic stimulation (TMS) already have negative effects on cognition as part of the disease process, thereby creating the possibility that treatment by TMS could further worsen mental symptoms already affected by the original disease process [7]. Despite these potential detrimental effects, long-term studies assessing the effects of TMS on cognition have, in fact, shown that repetitive sessions of s-TMS may leave unaltered [8], or even to some extent improve, cognitive channels (executive functions in particular) in patients suffering from neuropsychiatric diseases [9].

In the last 10 years, it has become clear that some neuropsychiatric conditions respond poorly to conventional s-TMS [10], while others do not respond at all [11]. For instance, bipolar mood disorders, Parkinson’s-disease-related motor symptoms, and some chronic pain syndromes such as central neuropathic pain have responded poorly to s-TMS, with either short-lasting effects [12] or clinically small effect sizes [10]. For these patients, new non-invasive cortical stimulation approaches have been proposed, such as deep-TMS (d-TMS). d-TMS allows for the stimulation of deeper cortical structures such as the dentate nucleus of the cerebellum [13], the insular [14] and the cingulate cortices [15], or the leg areas of the primary motor cortex [12, 16]. These structures participate in several disease processes and brain networks, and their non-invasive functional modulation creates the possibility of treating patients who have disease conditions not previously responsive to s-TMS.

In fact, several studies have assessed the effects of d-TMS on chronic neuropathic pain [15, 17], fibromyalgia [15], major depression [18], bipolar mood disorder [19], and Parkinson’s disease, thereby creating exciting potential treatment options for patients previously unresponsive to conventional s-TMS. Here, again, d-TMS has been shown to have a low risk of seizures so long as traditional safety guidelines concerning frequency and intensity of stimulation are followed [5, 20]. However, most current d-TMS studies were either single-session studies or studies with short-lasting stimulation periods (5–10 sessions, usually lasting less than 1–2 weeks) [21–29]. We have recently published the longest d-TMS study available to date, in which 98 patients with central neuropathic pain due to stroke or spinal cord injury were stimulated for a total of 16 sessions spanning a 12-week interval [30]. We found no effect of either posterior insular (PSI) or anterior cingulate cortex (ACC) compared to sham d-TMS on clinical pain, despite the finding of a significant anti-nociceptive on thermal thresholds after PSI d-TMS and a significant anxiolytic effect of ACC d-TMS compared to sham stimulation in these patients. Here, we report secondary outcome data from this trial on the results of a comprehensive evaluation of cognitive status performed before and after the long-term d-TMS treatment. This evaluation provides the largest and most comprehensive cognitive evaluation of the effects of multiple session d-TMS to date, and also provides supplementary information about the effects of d-TMS on the brains of patients with structural acquired central nervous system lesions, as half of the participants had stroke and its subsequent language and attentional deficits at baseline, while many of the spinal cord injury patients with central neuropathic pain had encephalic lesions due to demyelination or concomitant light to moderate traumatic brain injury related to the accident that produced the spinal cord injury (SCI) [31].

## Methods

The study was conducted at Hospital das Clínicas of the University of São Paulo, São Paulo, Brazil, from 2014 to 2017. Here, we present secondary outcome analyses focusing on the neuropsychological effects of treatment (long-term 12-week dTMS of the ACC, PSI, or sham in 98 patients with refractory central neuropathic pain where the primary outcome was pain intensity reduction after treatment). Our Ethics Review Board – Comissão de Ética para Análise de Projetos de Pesquisa– approved the protocol (#1.077.086) and all participants provided written informed consent before inclusion in the study. This study was registered at clinicaltrials.org. under the number NCT01932905.

### Patients

The subjects were adult patients diagnosed with central neuropathic pain [32] fulfilling diagnostic criteria from the International Association for the Study of Pain [33] due to spinal cord injury/inflammation or stroke, with a score of at least 40 on the pain intensity of the visual analogue scale (VAS) (0-100 mm) [34], and who gave informed consent to participate. Individuals who presented contraindications to magnetic resonance imaging or TMS, or who had a history of epilepsy or seizures, who had suffered a significant head injury in the 6 months preceding the study, or who had implanted ferromagnetic material, such as clips of an intracranial aneurysm and cardiac pacemaker, were excluded from the study [5]. Information about age, education, and pain duration was collected, as were scores for mood (Hospital Anxiety and Depression Scale) [35] at both the baseline and the end of the study (last day of stimulation). Patients currently in cognitive rehabilitation programs were not included in the study.

### Study design

**Fig. 1:**
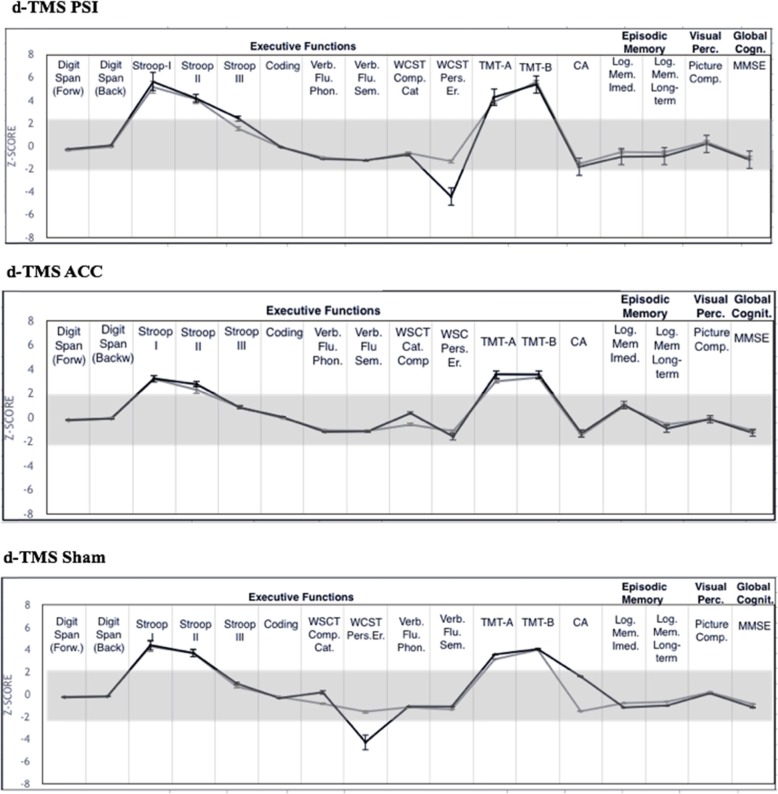
Effects of dTMS on cognitive tests

This was a randomized, three-arm, parallel, sham-controlled clinical trial. The randomization was performed through a random number generator (www.randomizer.com). Patients and raters were blinded to the treatment. Participants were randomly distributed into three groups: active stimulation of the right posterior superior insula (PSI-d-TMS), active stimulation of the right anterior cingulate cortex (ACC-d-TMS), or sham stimulation. The sham group was divided into the sham-PSI-d-TMS or the sham-ACC-d-TMS, as previously described [30]. Briefly, the stimulation protocol consisted of a total of 16 stimulation sessions spanning 12 weeks. Patients were stimulated for 5 consecutive days during the first week (induction phase) followed by a maintenance phase during which they received one stimulation weekly until the end of the study (Fig. 1). They were assessed at baseline and at the end of the stimulation program throughout the cognitive tests and scales to assess mood.

### Stimulation session

ACC d-T-MS was performed with a Magstim Rapid2 (Magstim Co, Ltd., Whitland Carmarthenshire, United Kingdom) and the PSI was stimulated with a cooled DB-80 double-cone coil (Magventure® Tonika-Elektronik, Farum, Denmark). Neuronavigation techniques were used to ensure the exact location of the insula. Sham d-™S PSI was performed through the use of an active figure-of-eight coil placed above the unplugged double-cone coil and the sham d-™S ACC a built-in sham system was used. In both types of stimulation, the noise that the coil emitted was the same. The determination of the rest motor threshold (RMT), the frequency used in the active stimulation, and other information has already been described [30]. All stimulations were performed on the right side at 10Hz (3000 pulses for 15 min).

### Clinical and mood assessment

Patients were assessed by a blinded investigator at baseline to explore sociodemographic characteristics and symptoms of anxiety and depression using the Hospital Anxiety and Depression Scale – (HADS). Each item is scored from 0 to 3 and an individual can score between 0 and 21 for either anxiety or depression, with 9 being the cut-off [35].

### Cognitive assessment

Patients were assessed at baseline and after the last session of treatment. The cognitive functions assessed included executive functions (attention, inhibitory control, processing speed, mental flexibility, verbal fluency [phonemic and semantic], and work memory) [36], episodic memory, global cognition, and visual perception [37]. The following cognitive domains and neuropsychological tests were used:

- *Global cognition:* Mini-Mental State Examination – MMSE, a 30-point questionnaire used to track cognitive loss [38]. The cut-off varies according to educational level.
- *Attention:*

Trails Making A/B – TMT A/B [39, 40], which has two parts, A and B. In the first part (A), the targets are numbers 1 to 25, written on a piece of paper. Individuals are asked to connect them in sequential order using a pen. In the second part (B), subjects are asked to connect numbers and letters of the alphabet sequentially (e.g., 1-A, 2-B, etc.). The score is based on the time that the subject takes to complete parts A and B. The process can last a maximum of 120 s for A and 300 s for B [41].

Concentrated Attention – CA, cancellation test to assess concentrated attention. The score ranges from 0 to 147 [42].

- *Inhibitory control*:
  - Victoria Stroop Test, which includes three conditions:
    - Naming block of color,
    - Naming ink color of words,
    - Naming ink color of incongruous color words [41].

The final score is the time taken to complete each part of the test in seconds.

ii. *Processing speed*:
  - Coding – The Wechsler Adult Intelligence Scale (Wais-III) involves sustained attention, focused concentration, and directed visual shifting. The score is the number of squares filled correctly in 2 min, and the maximum score is 133 [43].
iii. *Mental flexibility*: Wisconsin card sorting test (short form) (WSCT) modified version – A number of cards are presented and the subject is asked to match the cards, without further instruction. The subject is supposed to guess the “rule” of matching based on the rater’s positive or negative feedback (e.g., matching can be done by the geometric forms of the figures, by color, etc.). During the test, the “rule” is further changed and the individual is supposed to figure out what the new rule is. This test assesses mental flexibility. Errors and the completeness of each set are counted as positive results. The maximum score for each category is 6, while the maximum number of perseverative errors is 63 [44].
iv. *Verbal fluency (phonemic and semantic*):
  - Verbal fluency phonemic is a subtest of the *Neurosensory Center Comprehensive Examination for Aphasia* [41]. The individual is invited to speak words beginning with F, A, and S, taking 1 min for each letter, and semantic (animals), the subject must say animals in 1 min [41]. The score is a sum of the words in each part.
v. *Working memory*:
  - Digit Span – forward/backward – Wais-III [43], to evaluate a subject’s ability to store information in the short term and mentally manipulate it. The maximum score is 9 to forward and 8 to backward.
vi. *Episodic memory*: Subtest Logical Memory from the Wechsler Memory Scale WMS-R [45] evaluates immediate and long-term episodic memory through two stories that the examiner tells. The score can range from 0 to 50.
vii. *Visual perception*: Picture Completion – WAIS-III [43] is composed of figures with missing parts. The individual is expected to identify the missing part. The test assesses visual perception and the maximum score is 25.

### Statistical analysis

Results were expressed as median (minimum-maximal) values, as average +/−standard deviation, or as a 95% confidence interval depending on the nature of the data and the type of statistical test performed. To verify whether the distribution of the group’s results was normal, the Kolmogorov-Smirnoff test was used. As the distribution was not normal, non-parametric tests were used, such as the Kruskal-Wallis test followed by the Mann-Whitney test to compare two independent samples. To avoid baseline differences, deltas (Δ) were used based on the mean differences in calculation [(post-test – pre-test)/pre-test] for cognitive outcomes. Then, in a second analysis, patients’ scores were classified individually, based on published normative data [46], as “normal,” “low,” or “high.” These categorical variables were compared using the chi-square and McNemar tests. Also, scores from each subject were transformed into z-scores for the visual display of results. Scores were altered if they were 2 standard deviations (SD) below the reference data average (except for timed tests, in which performance was considered worse as the subject’s score increased) [41]. Spearman tests were used to explore correlations between cognitive scores and mood changes in the ACC-d-TMS group. A significance level of 0.05 was adopted and was then lowered according to Bonferroni’s correction. Only results that remained significant after correction for multiple comparisons were analyzed. All statistical analyses were carried out with SPSS, version 20.0 (SPSS, Inc., Chicago, 2009).

## Results

### Baseline assessment

One hundred patients (55.02 ± 12.13 years old, 45 females) were included in the study and were randomized and allocated to the respective treatment groups. Two dropped out of the study before the baseline assessment. Baseline demographic characteristics, etiology of central neuropathic pain, and pain location and characteristics were similar between groups at baseline and were reported elsewhere [30]. At baseline, cognitive scores did not significantly differ between groups, except for one of the subscores of the Stroop color interference test (i.e., Stroop effect), which was significantly lower in the ACC group (36.2 ± 18.1) compared to the PSI group (51.2 ± 27.1; *p* = 0.012) (Table 1). However, the proportion of patients with altered Stroop effect results at baseline did not differ between groups (Additional file 1: Table S1).

**Table 1:** Effects of dTMS to dTMS-PSI and dTMS-ACC groups compared to dTMS-Sham group

Baseline								After the last dTMS session (week 12)						
					Pairwise comparisons (*p*)							Pairwise comparisons (*p*)		
Cognitive Tests	dTMS-PSI (*n* = 33)	dTMS-ACC (*n* = 33)	dTMS-Sham (*n* = 32)	Inter group comparisons	dTMS -PSI X dTMS-ACC	dTMS-PSI X dTMS-Sham	dTMS-ACC X dTMS-Sham	dTMS-PSI (*n* = 33)	dTMS-ACC (*n* = 33)	dTMS-Sham (*n* = 32)	Inter group comparisons	dTMS- PSI X dTMS-ACC	PSI-dTMS X dTMS-Sham	dTMS-ACC X dTMS-Sham
Executive Functions				p#	p§						p#	p§		
Digit Span (forward)-Wais-III	4.4 ± 0.9	4.5 ± 1.1	4.4 ± 1.1	0.52	0.97	0.94	0.92	4.3 ± 0.94	4.53 ± 1.13	4.5 ± 0.88	0.44	0.61	0.55	0.85
Digit Span (backwards)-Wais-III	3.5 ± 0.9	3.4 ± 1.2	3.1 ± 0.9	0.20	0.31	0.07	0.51	3.3 ± 1.21	3.5 ± 1.51	3.2 ± 0.76	0.51	1.00	0.81	0.82
Stroop Test – I	25.6 ± 22	20 ± 8.3	23.4 ± 10.9	0.10	0.12	0.90	0.08	24.7 ± 14.55	19.9 ± 7.75	22.9 ± 11.63	0.56	0.35	0.95	0.31
Stroop Test – II	31.8 ± 19.7	26.3 ± 12.3	28.4 ± 13.7	0.07	0.14	0.45	0.33	31.1 ± 21.33	24.7 ± 13.93	28.8 ± 17.46	0.27	0.92	0.97	0.15
Stroop Test – III (Stroop effect)	51.2 ± 27.1	36.2 ± 18.1	40.2 ± 19.5	0.04*	0.012**	0.09	0.30	42.8 ± 21.72	36.5 ± 19.62	37 ± 16.11	0.73	0.25	0.68	0.72
Coding – Wais-III	33.2 ± 18.1	37.7 ± 20.3	27.7 ± 16.8	0.08	0.41	0.25	0.04*	33.7 ± 18.36	38.1 ± 18.43	30 ± 15.64	0.26	0.33	0.53	0.14
Verbal Fluency –Phon.	23.8 ± 10.2	23.8 ± 13	22.2 ± 9	0.34	0.87	0.54	0.77	25.2 ± 10.42	25.1 ± 15.43	21.6 ± 11.51	0.35	0.76	0.14	0.33
Verbal Fluency –Sem.	13.4 ± 5.2	13.8 ± 5.2	13.3 ± 4.4	0.88	0.68	0.97	0.53	13.3 ± 5.96	13.9 ± 5.01	12.2 ± 4.35	0.47	0.54	0.15	0.25
WCST-Perseverative errors	17.2 ± 12.8	17 ± 11.2	21.3 ± 12.3	0.14	0.85	0.12	0.06	15.2 ± 11.41	14.7 ± 10.23	17.3 ± 10.22	0.37	0.92	0.24	0.34
WCST-Completed categories	1.7 ± 1.5	2 ± 1.4	1.9 ± 1.6	0.53	0.39	0.68	0.74	2.3 ± 1.78	1.9 ± 1.50	1.7 ± 1.47	0.51	0.95	0.21	0.44
Trail Making A	80.1 ± 36.1	75.1 ± 14.3	80 ± 38.6	0.86	0.42	0.92	0.53	74.7 ± 48.51	67.3 ± 37.12	74.2 ± 34.22	0.33	0.57	0.62	0.21
Trail Making B	219.1 ± 102.1	180.1 ± 110.7	222.8 ± 167.9	0.18	0.08	0.39	0.33	232 ± 135.3	168.3 ± 108.3	217.1 ± 151.54	0.15	0.06	0.75	0.95
CA	38.2 ± 24.4	52 ± 31.4	41.8 ± 25.3	0.29	0.06	0.43	0.24	44.2 ± 28.13	49.5 ± 33.41	45.3 ± 28.21	0.44	0.62	0.93	0.93
Memory														
Logical Memory (I)-WMS III	16 ± 6.8	15.1 ± 8.3	14 ± 5.5	0.58	0.48	0.28	0.73	19.2 ± 7.46	18.1 ± 7.72	17.1 ± 7.12	0.61	0.86	0.21	0.31
Logical Memory (L)-WMSIII	10.7 ± 6.4	10.8 ± 8.9	9.2 ± 5.9	0.65	0.78	0.29	0.62	13.8 ± 72	13.4 ± 8.67	12.1 ± 7.51	0.76	0.83	0.22	0.53
Global Cognition														
MMSE	24.7 ± 3.1	24.8 ± 3.8	24.9 ± 3	0.88	0.77	0.79	0.70	24.9 ± 2.33	25.2 ± 3.92	25.5 ± 2.21	0.75	0.45	0.25	0.82
Visual Perception								11.5 ± 6.67	10.8 ± 6.64	10.2 ± 6.36	0.42	0.86	0.54	0.51
Picture Completion - Wais III	10.7 ± 5	9 ± 6	9.5 ± 5.8	0.29	0.08	0.40	0.79	11.5 ± 6.67	10.8 ± 6.64	10.2 ± 6.36	0.42	0.86	0.52	0.51

The values are presented as mean ± SD. p# = Kruskal Wallis between the 3 groups; p§ = Mann Whitney for 2 independent samples

*dTMS* Deep Transcranial Magnetic Stimulation, *PSI* Posterior Superior Insula, *ACC* Anterior Cingulate Cortex, *Wais-III* Wechsler Adult Intelligence Scale-III, *Phon* Phonemic, *Sem.* Semantic, *WCST* Wisconsin Card Sort Test, *CA* Concentrated Attention, (*I*) Immediate, (*L*) Long-term, *WMS III* Wechsler Memory Scale III, *MMSE* Mini Mental State Examination; * = *p* ≤ 0.05; ** = *p* ≤ 0.016 Bonferroni correction

### Effects of deep-TMS on cognitive function

There were no significant effects of stimulation on cognitive assessment scores during treatment. Median changes in scores in the active d-TMS groups were similar to those of the sham d-TMS group (Table 2) (*p* > 0.180) except for the verbal fluency – phonemic test, in which the d-TMS-PSI group showed a slight improvement (*p* = 0.025) after treatment; however, this change did not persist after corrections for multiple analyses (Additional file 1: Table S3).

**Table 2:** Mean differences [(post-test– pre-test)/pre-test] for cognitive outcomes

					Pairwise comparisons (*p*)		
Cognitive Tests	dTMS-PSI (*n* = 33)	dTMS-ACC (*n* = 33)	dTMS-Sham (*n* = 32)	Inter group comparisons	dTMS -PSI X dTMS-ACC	dTMS-PSI X dTMS-Sham	dTMS-ACC X dTMS-Sham
Executive Functions				p#	p§		
Digit Span (forward)-Wais-III	−0.08 ± 0.21	0.02 ± 0.16	0.04 ± 0.21	0.06	0.08	0.31	0.43
Digit Span(backward)-Wais-III	0.01 ± 0.47	0.09 ± 0.43	0.06 ± 0.31	0.44	0.65	0.17	0.53
Stroop Test – I	0.06 ± 0.52	0.008 ± 0.22	− 0.08 ± 0.20	0.93	0.80	0.86	0.69
Stroop Test – II	−0.01 ± 0.30	− 0.06 ± 0.18	0.04 ± 0.23	0.15	0.76	0.12	0.08
Stroop Test – III	−0.11 ± 0.25	0.00 ± 0.26	− 0.02 ± 0.23	0.54	0.33	0.35	0.96
Coding – Wais-III	0.11 ± 0.38	−0.06 ± 0.38	0.15 ± 0.42	0.32	0.16	0.91	0.22
Verbal Fluency –Phon.	0.14 ± 0.47	0.02 ± 0.31	0.05 ± 0.69	0.19	0.57	0.08	0.20
Verbal Fluency –Sem.	0.04 ± 0.38	0.02 ± 0.35	−0.02 ± 0.37	0.27	0.92	0.25	0.11
WCST-Perseverative errors	0.05 ± 0.79	−0.02 ± 0.55	− 0.05 ± 0.57	0.90	0.72	0.67	0.99
WCST-Completed categories	0.28 ± 0.97	0.2 ± 1.0	−0.22 ± 0.49	0.08	0.24	0.019*	0.33
Trail Making A	−0.05 ± 0.32	− 0.09 ± 0.39	−0.05 ± 0.31	0.68	0.51	0.75	0.43
Trail Making B	0.09 ± 0.49	−0.06 ± 0.41	−0.0004 ± 0.44	0.70	0.35	0.80	0.70
CA	0.22 ± 0.69	−0.10 ± 0.30	0.10 ± 0.55	0.07	0.02*	0.58	0.11
Memory							
Logical Memory(I)-WMS III	0.36 ± 0.97	0.28 ± 0.48	0.28 ± 0.52	0.94	0.92	0.69	0.85
Logical Memory(L)-WMSIII	0.48 ± 1.18	0.55 ± 1.04	0.41 ± 0.72	0.84	0.99	0.57	0.66
Global Cognition							
MMSE	0.02 ± 0.11	0.01 ± 0.09	0.03 ± 0.10	0.80	0.94	0.51	0.62
Visual Perception							
Picture Completion. - Wais III	0.09 ± 0.42	0.33 ± 0.62	0.15 ± 0.52	0.45	0.26	0.94	0.29

The values are presented as mean ± SD. p# = Kruskal-Wallis test; p§ = U Mann Whitney test

*dTMS* Deep Transcranial Magnetic Stimulation, *PSI* Posterior Superior Insula, *ACC* Anterior Cingulate Cortex, *Wais-III* Wechsler Adult Intelligence Scale, *Phon.* Phonemic, *Sem.* Semantic, *WCST* Wisconsin Card Sort Test, *CA*, Concentrated Attention, (*I*) Immediate, (*L*) Long-term, *WMS III* Wechsler Memory Scale III, *MMSE* Mini Mental State Examination; * = *p* ≤ 0.05; ** = *p* ≤ 0.016 Bonferroni correction

In an individual-based analysis, the proportion of scores classified as low, normal, or high according to normative data failed to show significant differences between groups after treatment with d-TMS (results only provided in the Additional file 1: Table S2).

As previously reported, while active stimulation did not influence depression symptoms, anxiety subscores of the HADS were significantly reduced by active ACC-d-TMS (− 2.96 95%CI [− 4.1; − 1.7]) compared to sham d-TMS (− 0.78 95%CI [− 1.9; 0.3] *p* = 0.018) and PSI-d-TMS (0.15 95%CI[− 1.0; 1.3]) arms, respectively [30]. However, there were no significant correlations between changes in each of the neuropsychological tests and anxiety improvement after Bonferroni’s correction.

## Discussion

In this randomized, sham controlled study, we have found no negative cognitive effects of d-TMS targeted to the ACC and PSI in a large sample of patients with CNS lesions. Our data suggest that high-frequency deep-TMS to these structures was safe and well-tolerated.

In the present study, patients underwent a comprehensive battery of neurocognitive tests, including working and episodic memory, inhibitory control, attention, mental flexibility, processing speed, verbal fluency (phonemic and semantic), global cognition evaluation, and visual perception assessment. We have compared scores at baseline and after a full induction and maintenance run of active d-TMS against a sham stimulation. We have also performed a supplementary analysis in which each patient was classified as having either a normal, low, or high score based on published reference data. This analysis reported on and compared the percentage of altered results in each group. We found that despite the occurrence of clear biological effects of active PSI and ACC d-TMS on sensory thresholds and anxiety, respectively [30], there were no changes in cognitive functioning after d-TMS treatment. While several studies have reported on the safety of s-TMS in psychiatric and neurological disorders [9, 47–49], few have included more than 25 patients per arm [9, 50–52], and the vast majority of them evaluated the effects of s-TMS targeted to the dorsolateral prefrontal cortex [47–49, 51, 53–61], as this is a common superficial target in major depression treatment trials. Deeper brain areas – particularly cortices located deeply within the brain parenchyma – have seldom been assessed [29].

Presently, only a few trials have used d-TMS and reported outcomes for at least one neuropsychological [21–29] score. These trials included healthy subjects [23–26], individuals with memory complaints [21], or patients affected by psychiatric disorders such as major depression [24, 25], schizophrenia [27], and obsessive-compulsive disorder [28]. Total study sample sizes ranged from 10 to 35. Except for four studies [23, 28, 62], all the remaining used the “hesed” (H) coils aimed at targets located within the frontal lobe. Among these studies, only five included multiple sessions of stimulation days extending beyond a week [24, 25, 27–29]. The others reported results after 1–5 sessions of d-TMS only [22, 23, 26, 62]. In the totality of trials, stimulation was performed for a maximum of 4 weeks. Importantly, none of the trials included patients who had structural brain lesions and who already had major cognitive deficits and increased risk of seizures at baseline, such as those included here. Concerning the results, while some reports have claimed improvement in declarative memory [62] and spatial working memory [25, 28] in healthy individuals and in bipolar depression patients [29], or transient aggravation of spatial recognition memory [22] and mental flexibility [28], most of them have found no changes in [23, 25–27] cognitive functions. It must, however, be kept in mind that with rare exceptions [26, 29], cognitive assessments were often limited to a few cognitive domains and were not comprehensive enough.

We have here, for the first time, reported on the cognitive effects of the high frequency of two deep cortical targets: the right PSI and the right ACC. Resting-state studies have shown bilateral ventrolateral prefrontal cortex and anterior insular cortex activation (and ACC activation coupled to activation in the amygdala and hypothalamus) – a pattern that has been called the “salience network” (SN). This large-scale network integrates the internal, external, and memory information with the aim of directing attention towards behaviorally relevant inputs [63]. The SN is strongly lateralized to the right anterior insular cortex, which connects this network with two other large networks: the central executive network (CEN) and the default mode network (DMN). While the CEN is composed of large areas of the (mainly left) dorsolateral prefrontal and posterior parietal cortices and is related to executive functions, the DMN is composed of medial prefrontal-posterior cingulate cortex connections and is related to mind wandering and interoception. The SN is then at a strategic point where it controls the shifting of attention from internal inputs to attention-demanding cognitive tasks. In fact, impaired temporal fluctuation of the DMN is common in major depression and bipolar mood disorders. Thus, so far, most studies on dTMS have targeted the dorsalateral prefrontal cortex and neighbor regions, frequently related to CEN, while none have assessed the cognitive effects of direct stimulation of SN structures as done here over the right ACC. We have shown here that despite the presence of biological effects of ACC and PSI (on anxiety and nociception, respectively), no changes were observed in any of the many cognitive domains assessed. In fact, we found no significant correlations between cognitive changes in neuropsychological tests and improvement in anxiety. This is an important issue because Levkovitz et al. (2009) have previously reported that improvement in anxiety and attention, as well as in memory and psychomotor speed, may occur after s-TMS to the dorsolateral prefrontal cortex. Also, the presence of pain is known to negatively affect cognition, especially executive functions. This is probably related to the fact that chronic pain affects the connectivity between the SN and DMN, decreasing the normal shift of attention to and from the different tasks needed for normal cognitive functioning [64]. In the present study, all patients had refractory central neuropathic pain and were under treatment with psychoactive drugs. Because all groups took a similar number of drugs and had similar doses of medication, and because the different d-TMS treatment regimens were ineffective at reducing pain, we believe these variables had little effects on our results. However, one cannot attest that subtler cognitive effects of d-TMS to the right PSI or ACC were not missed secondary to “noise” induced by the use of psychotropic analgesics. Also, due to the ceiling effect, one cannot exclude the notion that deep-TMS to the ACC and PSI would not cause cognitive impairment in healthy individuals, as they were not assessed here. Thus, the relative safety of the procedure concerns patients with structural CNS lesions.

## Conclusion

This study confirms that 16 sessions of deep-TMS in PSI and CCA at a frequency of 10 Hz and 80% of MT have no deleterious effect on cognitive functioning in patients with neuropathic pain. This study indicates that deep brain stimulation is safe even in the presence of cognitive impairment at baseline and structural brain diseases. However, further studies using this technique are necessary to test whether other stimulation parameters can exert positive effects on chronic pain and to assess whether other types of stimulation (e.g., patterned theta-burst stimulation) [65] have this same safety profile.

## Additional file

**Additional file 1: Table S1.** Individual classification of patients scores based on normative data at baseline. **Table S2.** Distribution of the sample according to the results of the tests classified according to normative tables in absolute numbers, percentage and results of test of association (*p*) of the groups dTMS-PSI, dTMS-ACC and dTMS-Sham at the post - treatment (T1). **Table 3.** Comparison of cognitive test results between pre and post treatment, classified from normative tables in absolute numbers, percentage and association test results (*p*) of the dTMS-PSI; dTMS-ACC and dTMS -Sham groups.

## Data Availability

The datasets used and analyzed during the current study are available upon request to the corresponding author.

## Abbreviation

ACC: Anterior cingulate cortex
CA: Concentrated Attention
CEN: Central Executive Network
CNS: Central nervous system
DMN: Default mode network
d-TMS: Deep transcranial magnetic stimulation
HADS: Hospital Anxiety and Depression Scale
MMSE: Mini Mental State Examination
PSI: Posterior insula
RMT: Rest motor threshold
rTMS: Repetitive transcranial magnetic stimulation
SCI: Spinal cord injury
SN: Salience Network
s-TMS: Superficial transcranial magnetic stimulation
TMS: Transcranial magnetic stimulation
TMT: Trail Making
VAS: Visual analogue scale
WAIS-III: Wechsler Adult Intelligence Scale
