# Supplementary material for: Long-term deep-TMS does not negatively affect cognitive functions in stroke and spinal cord injury patients with central neuropathic pain
Source: BMC Neurol. 2019 Dec 10;19:319. doi: 10.1186/s12883-019-1531-z (PMC6905077; doi:10.1186/s12883-019-1531-z)
Supplement: Supplementary file 1 — Additional file 1: Table S1. Individual classification of patients scores based on normative data at baseline. Table S2. Distribution of the sample according to the results of the tests classified according to normative tables in absolute numbers, percentage and results of test of association (p) of the groups dTMS-PSI, dTMS-ACC and dTMS-Sham at the post - treatment (T1). Table 3. Comparison of cognitive test results between pre and post treatment, classified from normative tables in absolute numbers, percentage and association test results (p) of the dTMS-PSI; dTMS-ACC and dTMS -Sham groups. [file 12883_2019_1531_MOESM1_ESM.docx]

**Table S1** – Individual classification of patients scores based on normative data at baseline.

| **Cognitive Tests** | **dTMS -PSI** | | | **dTMS -ACC** | | | | **dTMS-Sham** | | | | **p** |
| --- | --- | --- | --- | --- | --- | --- | --- | --- | --- | --- | --- | --- |
| **Executive Functions** | **Low** | **Normal** | **High** | | **Low** | **Normal** | **High** | | **Low** | **Normal** | **High** |  |
| Digit Span (forward)-Wais III | 4(12.1%) | 28(84.8%) | 1(3%) | | 2(6.1%) | 28(84.8) | 3(9.1%) | | 3(9.4%) | 28(87.5%) | 1(3.1%) | 0.693 |
| Digit Span (backward)-Wais III | 0(0%) | 33(100%) | 0(0%) | | 0(0%) | 32(97%) | 1(3%) | | 0(0%) | 32(100%) | 0(0%) | 0.370 |
| Stroop Test – I | 23(69.7%) | 8(24.2%) | 0(0%) | | 16(48.5%) | 14(42.4%) | 0(0%) | | 21(65.6%) | 8(25%) | 0(0%) | 0.419 |
| Stroop Test – II | 22(66.7%) | 9(27.3%) | 0(0%) | | 15(45.5%) | 15(45.5%) | 0(0%) | | 16(50%) | 12(37.5%) | 0(0%) | 0.442 |
| Stroop Test – III (Stroop effect) | 13(39.4%) | 18(54.5%) | 0(0%) | | 6(18.2%) | 24(72.7%) | 0(0%) | | 6(18.8%) | 22(68.8%) | 0(0%) | 0.247 |
| Coding – Wais III | 0(0%) | 32(97%) | 0(0%) | | 1(3%) | 27(81.8%) | 0(0%) | | 0(0%) | 29(90.6%) | 0(0%) | 0.284 |
| Verbal Fluency –Phon. | 6(18.2%) | 27(81.8%) | 0(0%) | | 4(12.1%) | 26(78.8%) | 1(3%) | | 2(6.2%) | 30(93.8%) | 0(0%) | 0.219 |
| Verbal Fluency –Sem. | 8(24.2%) | 25(75.8%) | 0(0%) | | 9(27.3%) | 23(69.7%) | 0(0%) | | 5(15.6%) | 27(84.4%) | 0(0%) | 0.480 |
| WCST-Perseverative errors | 4(12.1%) | 28(84.8%) | 0(0%) | | 4(12.1%) | 27(81.8%) | 0(0%) | | 2(6.2%) | 28(87.5%) | 0(0%) | 0.876 |
| WCST-Completed categories | 26(78.8%) | 6(18.2%) | 0(0%) | | 22(66.6%) | 9(27.3%) | 0(0%) | | 22(68.8%) | 8(25%) | 0(0%) | 0.838 |
| Trail Making A | 22(66.7%) | 10 (30%) | 0(0%) | | 14(42.4%) | 14(42.4%) | 0(0%) | | 17(53.1%) | 13(40.6%) | 0(0%) | 0.220 |
| Trail Making B | 23(69.7%) | 8(24.2%) | 0(0%) | | 16(48.5%) | 12(36.4%) | 0(0%) | | 17(53.1%) | 11(34.4%) | 0(0%) | 0.463 |
| CA | 22(66.7%) | 10(30.3%) | 0(0%) | | 13(39.4%) | 16(48.5%) | 0(0%) | | 16(50%) | 14(43.8%) | 0(0%) | 0.215 |
| **Memory** |  |  |  |  |  |  |  |  |  |  |  |  |
| Logical Memory (I)-WMS III | 5(15.2%) | 28(84.8%) | 0(0%) | | 7(21.2%) | 26(78.8%) | 0(0%) | | 3(9.4%) | 29(90.6%) | 0(0%) | 0.415 |
| Logical Memory (L)-WMS III | 4(12.1%) | 29(87.9%) | 0(0%) | | 7(21.2%) | 26(78.8%) | 0(0%) | | 5(15.6%) | 27(84.4%) | 0(0%) | 0.602 |
| **Visual Perception** |  |  |  |  |  |  |  |  |  |  |  |  |
| Picture Completion. - Wais III | 0(0%) | 31(93.9%) | 1(3%) | | 1(3%) | 31(93.9%) | 0(0%) | | 0(0%) | 30(93.8%) | 0(0%) | 0.611 |
| **Global Cognition** |  |  |  |  |  |  |  |  |  |  |  |  |
| MMSE | 11(33.3%) | 22(66.7%) | 0(0%) | | 11(33.3%) | 22(66.7%) | 0(0%) | | 11(34.4%) | 20(62.5%) | 0(0%) | 0.712 |

Results are expressed as absolute number of patients (%) with low, normal or high scores, respectively. p=Chi-square test.

Legends: dTMS= deep Transcranial Magnetic Stimulation; PSI= Posterior Superior Insula; ACC= Anterior Cingulate Cortex; Wais III = Wechsler Adult Intelligence Scale; Phon.= Phonemic; Sem.= Semantic; WCST= Wisconsin Card Sort Test; CA: Concentrated Attention, (I)= Immediate; (L)= Long-term; WMS III= Wechsler Memory Scale III; MMSE= Mini Mental State Examination; *p ≤ 0.05; **p ≤ 0.016 for Bonferroni correction.

**Table S2** - Distribution of the sample according to the results of the tests classified according to normative tables in absolute numbers, percentage and results of test of association (p) of the groups dTMS-PSI, dTMS-ACC and dTMS-Sham at the post - treatment (T1)

| **Cognitive Tests** | | | **dTMS -PSI** | | | | | | | | | | | | | | | **dTMS -ACC** | | | **dTMS-Sham** | | | | **p** | | |
| --- | --- | --- | --- | --- | --- | --- | --- | --- | --- | --- | --- | --- | --- | --- | --- | --- | --- | --- | --- | --- | --- | --- | --- | --- | --- | --- | --- |
| **Executive Functions** | | | **Low** | | | | | | | | **Normal** | | | | | **High** | **Low** | | **Normal** | **High** | | **Low** | **Normal** | **High** | |  | |
| Digit Span (forward)-Wais III | | | | | | 4(12.1%) | | | | | 28(84.8%) | | | | | 1(3%) | 2(6.1%) | | 28(84.8) | 3(9.1%) | | 3(9.4%) | 28(87.5%) | 1(3.1%) | | 0.693 | |
| Digit Span (backward)-Wais III | | | | | | 0(0%) | | | | | 33(100%) | | | | | 0(0%) | 0(0%) | | 32(97%) | 1(3%) | | 0(0%) | 32(100%) | 0(0%) | | 0.370 | |
| Stroop Test – I | | | | | | 23(69.7%) | | | | | 8(24.2%) | | | | | 0(0%) | 16(48.5%) | | 14(42.4%) | 0(0%) | | 21(65.6%) | 8(25%) | 0(0%) | | 0.419 | |
| Stroop Test – II | | | | | | 22(66.7%) | | | | | 9(27.3%) | | | | | 0(0%) | 15(45.5%) | | 15(45.5%) | 0(0%) | | 16(50%) | 12(37.5%) | 0(0%) | | 0.442 | |
| Stroop Test – III | | | | | | 13(39.4%) | | | | | 18(54.5%) | | | | | 0(0%) | 6(18.2%) | | 24(72.7%) | 0(0%) | | 6(18.8%) | 22(68.8%) | 0(0%) | | 0.247 | |
| Coding – Wais III | | | | | | 0(0%) | | | | | 32(97%) | | | | | 0(0%) | 1(3%) | | 27(81.8%) | 0(0%) | | 0(0%) | 29(90.6%) | 0(0%) | | 0.284 | |
| Verbal Fluency –Phon. | | | | | | 6(18.2%) | | | | | 27(81.8%) | | | | | 0(0%) | 4(12.1%) | | 26(78.8%) | 1(3%) | | 2(6.2%) | 30(93.8%) | 0(0%) | | 0.219 | |
| Verbal Fluency –Sem. | | | | | | 8(24.2%) | | | | | 25(75.8%) | | | | | 0(0%) | 9(27.3%) | | 23(69.7%) | 0(0%) | | 5(15.6%) | 27(84.4%) | 0(0%) | | 0.480 | |
| WCST-Perseverative errors | | | | | | 4(12.1%) | | | | | 28(84.8%) | | | | | 0(0%) | 4(12.1%) | | 27(81.8%) | 0(0%) | | 2(6.2%) | 28(87.5%) | 0(0%) | | 0.876 | |
| WCST-Completed categories | | | | | | 26(78.8%) | | | | | 6(18.2%) | | | | | 0(0%) | 22(66.6%) | | 9(27.3%) | 0(0%) | | 22(68.8%) | 8(25%) | 0(0%) | | 0.838 | |
| Trail Making A | | | | | | 22(66.7%) | | | | | 10 (30%) | | | | | 0(0%) | 14(42.4%) | | 14(42.4%) | 0(0%) | | 17(53.1%) | 13(40.6%) | 0(0%) | | 0.220 | |
| Trail Making B | | | | | | 23(69.7%) | | | | | 8(24.2%) | | | | | 0(0%) | 16(48.5%) | | 12(36.4%) | 0(0%) | | 17(53.1%) | 11(34.4%) | 0(0%) | | 0.463 | |
| CA | | | | | | 22(66.7%) | | | | | 10(30.3%) | | | | | 0(0%) | 13(39.4%) | | 16(48.5%) | 0(0%) | | 16(50%) | 14(43.8%) | 0(0%) | | 0.215 | |
| **Memory** |  |  | |  |  | | | | | | | | | | | | | | | | | | | | | |  |
| Logical Memory (I)-WMS III | | | | | | 5(15.2%) | | | | | 28(84.8%) | | | | | 0(0%) | 7(21.2%) | | 26(78.8%) | 0(0%) | | 3(9.4%) | 29(90.6%) | 0(0%) | | 0.415 | |
| Logical Memory (L)-WMS III | | | | | | 4(12.1%) | | | | | 29(87.9%) | | | | | 0(0%) | 7(21.2%) | | 26(78.8%) | 0(0%) | | 5(15.6%) | 27(84.4%) | 0(0%) | | 0.602 | |
| **Visual Perception** | | | | | |  | |  | |  | | |  | |  |  |  |  |  |  |  |  |  |  |  |  |  |
| Picture Completion - Wais III | | | | | | 0(0%) | | | | | 31(93.9%) | | | | | 1(3%) | 1(3%) | | 31(93.9%) | 0(0%) | | 0(0%) | 30(93.8%) | 0(0%) | | 0.611 | |
| **Global Cognition** | | | | | |  |  | |  | | |  | |  |  |  |  |  |  |  |  |  |  |  |  |  |  |
| MMSE | | | | | | 11(33.3%) | | | | | 22(66.7%) | | | | | 0(0%) | 11(33.3%) | | 22(66.7%) | 0(0%) | | 11(34.4%) | 20(62.5%) | 0(0%) | | 0.712 | |

Results are expressed as absolute number of patients (%) with low, normal or high scores, respectively. p=Chi-square test.

Legends: dTMS= deep Transcranial Magnetic Stimulation; PSI= Posterior Superior Insula; ACC= Anterior Cingulate Cortex; Wais III = Wechsler Adult Intelligence Scale -III; Phon.= Phonemic; Sem.= Semantic; WCST= Wisconsin Card Sort Test; CA: Concentrated Attention, (I)= Immediate; (L)= Long-term; WMS III= Wechsler Memory Scale III; MMSE= Mini Mental State Examination; *p ≤ 0.05; **p ≤ 0.016 for Bonferroni correction.

**Table S3** – Comparison of cognitive test results between pre and post treatment, classified from normative tables in absolute numbers, percentage and association test results (p) of the dTMS-PSI; dTMS-ACC and dTMS -Sham groups.

| **Cognitive Tests** | **dTMS -PSI** | | | | **dTMS-ACC** | | | | | **dTMS-Sham** | | | | | | | | |  |
| --- | --- | --- | --- | --- | --- | --- | --- | --- | --- | --- | --- | --- | --- | --- | --- | --- | --- | --- | --- |
| **Executive Functions** | **T0** | | **T1** | | **p** | **T0** | | **T1** | | | **p** | | **T0** | | **T1** | | | **p** |  |
|  | **Normal** | **Changed** | **Normal** | **Changed** |  | **Normal** | **Changed** | **Normal** | **Changed** | |  | **Normal** | | **Changed** | | **Normal** | **Changed** |  | |
| Digit Span (forward)-Wais III | 28(84.8%) | 5(15.2%) | 29(87.9%) | 4(12.1%) | 1.000 | 27(81.8%) | 5(15.2%) | 30(91%) | 2(6.2%) | | 0.375 | 28(87.5%) | | 4(12.5%) | | 30(93.8%) | 2(6.2%) | 0.687 | |
| Digit Span(backward)-Wais III | 30(91%) | 3(9%) | 30(91%) | 3(9%) | 1.000 | 31(93.9%) | 1(3%) | 27(84.4%) | 5(15.1%) | | 0.125 | 32(100%) | | 0(0%) | | 32(100%) | 0(0%) | 1000 | |
| Stroop Test – I | 32(97%) | 0(0%) | 32(97%) | 0(0%) | 1.000 | 27(84.4%) | 0(0%) | 26(78.7%) | 0(0%) | | 1.000 | 26(81.2%) | | 0(0%) | | 26(81.2%) | 0(0%) | 1.000 | |
| Stroop Test – II | 25(75.7%) | 8(24.2%) | 24(72.7%) | 9(27.2%) | 1.000 | 22(67%) | 9(27%) | 25(76%) | 6(18%) | | 0.180 | 27(84%) | | 5(16%) | | 26(81%) | 6(19%) | 1.000 | |
| Stroop Test – III | 27(82%) | 6(18%) | 29(88%) | 4(12%) | 0.500 | 27(84.4%) | 3(9.4%) | 25(78.1%) | 5(5.6%) | | 0.157 | 30(94%) | | 2(6%) | | 24(75%) | 8(25%) | **0.031*** | |
| Coding – Wais III | 8(24%) | 23(69.6%) | 10(30%) | 21(64%) | 0.317 | 14(42.4%) | 15(46.9%) | 15(45.4%) | 14(42.4%) | | 0.705 | 8(25%) | | 21(65.6%) | | 8(25%) | 21(65.6%) | 1.000 | |
| Verbal Fluency –Phon. | 9(27%) | 22(66.7%) | 9(27%) | 22(66.7%) | 1.000 | 15(45.4%) | 14(43.8%) | 16(48%) | 13(40.6%) | | 0.655 | 12(37.5%) | | 16(50%) | | 11(37.5%) | 17(53.1%) | 0.549 | |
| Verbal Fluency –Sem. | 18(54%) | 13(39%) | 23(69.6%) | 8(24%) | **0.025*** | 23(72.7%) | 6(18%) | 22(68.8%) | 7(21%) | | 0.655 | 21(69%) | | 6(18.7%) | | 22(68.7%) | 5(15.6%) | 0.368 | |
| WCST-Perseverative errors | 28(84.8%) | 4(12%) | 29(87.8%) | 3(9%) | 0.317 | 26(78.7%) | 4(12%) | 27(81.8%) | 3(9%) | | 0.564 | 28(87.5%) | | 2(6.2%) | | 27(84.3%) | 3(9%) | 0.317 | |
| WCST-Completed categories | 6(18%) | 26(79%) | 8(24%) | 24(72.7%) | 0.414 | 9(27%) | 21(65.6%) | 9(27%) | 21(63.6%) | | 1.000 | 8(25%) | | 22(68.7%) | | 5(15.6%) | 25(78,1%) | 0.180 | |
| Trail Making A | 10(30.3%) | 22(66.6%) | 15(45.4%) | 17(51.5%) | 0.059 | 14(42.4%) | 13(42.4%) | 13(39.3%) | 14(42.4%) | | 1.000 | 13(40.6%) | | 16(51.6%) | | 12(37.5%) | 17(53.1%) | 0.655 | |
| Trail Making B | 8(24%) | 23(69.6%) | 9(27%) | 22(67%) | 0.564 | 12(36%) | 14(42.9%) | 13(39%) | 13(39%) | | 0.705 | 11(34.3%) | | 16(50%) | | 11(34.3%) | 16(50%) | 1.000 | |
| CA | 10(30.3%) | 22(66.6%) | 14(42.4%) | 18(54.4%) | 0.102 | 16(48.4%) | 12(37.5%) | 11(33.3%) | 17(51.5%) | | **0.025*** | 12(37.5%) | | 16(50%) | | 11(37.5%) | 28(87.5%) | 0.549 | |
| **Memory** |  |  |  |  |  |  |  |  |  | |  |  | |  | |  |  |  | |
| Logical Memory (I)-WMS III | 28(84,5%) | 5(15%) | 30(91%) | 3(9%) | 0.625 | 25(75.7%) | 7(21.9%) | 28(87.5%) | 4(12.5%) | | 0.250 | 29(90,6%) | | 3(9.3%) | | 29(90.6%) | 3(9.4%) | 1.000 | |
| Logical Memory (L)-WMS III | 29(87.9%) | 4(12.1%) | 31(94%) | 2(6%) | 0.625 | 25(75.7%) | 6(18.1%) | 28(84.8%) | 3(9%) | | 0.250 | 27(84,3%) | | 5(15.6%) | | 28(87.5%) | 4(12.5%) | 1.000 | |
| **Visual Perception** |  |  |  |  |  |  |  |  |  | |  |  | |  | |  |  |  | |
| Picture Completion - Wais III | 25(75.7%) | 6(18.1%) | 28(84.8%) | 3(9%) | 0.250 | 31(93.9%) | 0(0%) | 31(93.9%) | 0(0%) | | 1.000 | 30(87,5%) | | 0(0%) | | 30(93,7%) | 0(0%) | 1,000 | |
| **Global Cognition** |  |  |  |  |  |  |  |  |  | |  |  | |  | |  |  |  | |
| MEEM | 22(66.7%) | 11(33.3%) | 20(61%) | 13(39%) | 0.727 | 21(66.7%) | 11(33.3%) | 19(57.5%) | 13(39.4%) | | 0.754 | 20(62.5%) | | 11(34.3%) | | 22(68.7%) | 9(28.1%) | 0.480 | |

Results are expressed as absolute number of patients (%) with normal or changed, respectively. p= McNemar test.

Legends: dTMS= deep Transcranial Magnetic Stimulation; PSI= Posterior Superior Insula; ACC= Anterior Cingulate Cortex; Wais III = Wechsler Adult Intelligence Scale -III; Phon.= Phonemic; Sem.= Semantic; WCST= Wisconsin Card Sort Test; CA: Concentrated Attention, (I)= Immediate; (L)= Long-term; WMS III= Wechsler Memory Scale III; MMSE= Mini Mental State Examination; *p ≤ 0.05; **p ≤ 0.016 for Bonferroni correction.
